# Formulating a researchable question: A critical step for facilitating good clinical research

**DOI:** 10.4103/0253-7184.69003

**Published:** 2010

**Authors:** Sadaf Aslam, Patricia Emmanuel

**Affiliations:** Clinical and Translational Science Institute and Department of Internal Medicine, College of Medicine, University of South Florida, Tampa, FL, USA

**Keywords:** Clinical research project, PICO format, research question

## Abstract

Developing a researchable question is one of the challenging tasks a researcher encounters when initiating a project. Both, unanswered issues in current clinical practice or when experiences dictate alternative therapies may provoke an investigator to formulate a clinical research question. This article will assist researchers by providing step-by-step guidance on the formulation of a research question. This paper also describes PICO (population, intervention, control, and outcomes) criteria in framing a research question. Finally, we also assess the characteristics of a research question in the context of initiating a research project.

## INTRODUCTION

A researchable question is an uncertainty about a problem that can be challenged, examined, and analyzed to provide useful information.[1] A successful research project depends upon how well an investigator formulates the research question based on the problems faced in day-to-day research activities and clinical practice. The underlying questions of a research project provide important information to decide whether the topic is relevant, researchable, and significant. A well-formulated research question needs extreme specificity and preciseness which guides the implementation of the project keeping in mind the identification of variables and population of interest. Here we will present a clinical scenario and see how clinical questions arise and help us in finding the evidence to answer our question.

## FORMULATING THE RESEARCH QUESTION

### Case

A 2-year-old boy presents in an outpatient clinic with fever and severe pain in his right ear. He has a history of recurrent ear infections, and his mother expresses a concern that he has been on the antibiotic amoxicillin for the past few weeks. She is worried about the consequences of the long-term antibiotic use. She is also concerned about the outcome associated with recurrent ear infections. She wants to know if the prescribed amoxicillin is effective, or it can be substituted with another antibiotic because of its side effects such as frequent diarrhea.

Several questions arise from this case which can be broadly classified into background and foreground questions. The general questions about a clinical problem or a disease are called “Background Questions.”[2] These questions generally ask what, when, how, and where about the disease, disorder, or treatment for instance, “What is otitis media?” or “How does amoxicillin work?” etc. These types of questions can be answered by going through review articles or text books.

The patient-oriented questions involving interpretation of a therapy or disease and consideration of risk vs. benefit for a patient or a group of patient are called “Foreground Questions.”[2] These types of complex clinical questions are best answered by primary or pre-assessed studies in the literature. These questions mostly compare the two, either two drugs or treatments or two diagnostic methods, etc.

The PICO (population, intervention, control, and outcomes) format [Table 1] is considered a widely known strategy for framing a “foreground” research question.[3] Sackett *et al*. pointed out that breaking the question into four components will facilitate the identification of relevant information.

**Table 1:** Considering PICO and FINER criteria for developing a research question[3,5]

*PICO*	
P: Population of interest	Patient or the problem to be addressed
I: Intervention	Exposure to be considered–treatments/ tests
C: Control	Control or comparison intervention treatment/placebo/standard of care
O: Outcome	Outcome of interest
*FINER*	

F: Feasibility	Suffi cient resources in terms of time, staff, and funding Use of appropriate study design Manageable in scope Adequate sample size Trained research staff
I: Interesting	Interesting as a researcher or collaborator Investigator’s motivation to make it interesting
N: Novel	Thorough literature search New fi ndings or extension of previous findings Guidance from mentors and experts
E: Ethical	Following ethical guidelines Regulatory approval from Institutional Review Board
R: Relevant	Influence on clinical practice Furthering research and health policy

*Population or problem*- addressing a specific population, its important characteristics and demographic information. From the above case, you can identify pediatric population with otitis media, the age range, sex, presenting complaint, and history.

*Intervention or treatment of interest*- the intervention can be a treatment, procedure, diagnostic test, and risk or prognostic factors. In this case, the intervention will be your plan to treat the patient which can be a new therapy, a diagnostic test, prognostic factor, or a procedure. For example, based on your observation in clinic, cefuroxime is another better treatment option as compared to amoxicillin in treating otitis media but you are not sure about its efficacy in pediatric population with otitis media.

*Comparator or control*-when a new therapy is compared with the existing one.

*Outcome*- is the effect of the intervention. For example, its effectiveness in controlling pain. Therefore, the outcome in the above case can be the relief of pain, the resolution of infection, or decreasing the risk of developing resistance. A good primary outcome should be easily quantifiable, specific, valid, reproducible, and appropriate to your research question.[4]

In a typical clinical setting, a clinician needs to know about background and foreground questions depending upon the experience about a particular disease and therapy. Once background questions are answered, more complex questions are addressed. The clinical questions arise from the central issues in a clinical work.[2] For example, identifying causes or risk factors (etiological questions), comparing diagnostic tests based on sensitivity and specificity (diagnostic query), identifying best treatment options (therapeutic question), and outcome of the treatment (prognostic question).

After determining a foreground question, the PICO approach is followed. Dissecting the question into parts makes it easy and searchable. As evident in this case, there are several relevant questions, for example: what are the outcomes associated with recurrent ear infection, what are the possible effects of long-term use of antibiotic, and what are the harms associated with current treatment? Now if you gather all the information from PICO approach, the following researchable questions can be formulated.

In children with acute otitis media (P), is cefuroxime (I) effective in reducing the duration of symptoms (O) as compared to amoxicillin (C)?

In children suffering from otitis media, will cefuroxime result in the improvement of symptoms and reduction in developing resistance?

Does treatment with amoxicillin increase the risk of developing resistance in children suffering from otitis media?

Does surgical procedure has better outcome for the treatment of otitis media in children after repeated antibiotic therapy?

From the above case, we have formulated multiple questions based on our patient’s illness and concerns. Now we can use the strategy of “selecting” the best question.[2] For example, which question has more significance for the patient’s well-being, which question is relevant to our knowledge needs and which question might lead to interesting answers for our patients and clinical query? Further, we need to consider the feasibility of finding the evidence in a short period.

## ASSESSING THE RESEARCH QUESTION IN THE CONTEXT OF A STUDY DESIGN

As proposed by Hulley *et al*. [Table 1], a research question should be formulated keeping in mind the FINER (feasible, interesting, novel, ethical, and relevant) criteria[5] and that the answer should fill gaps in the existing knowledge. The following points should be considered while assessing a research question.

Determining the required resources

The feasibility of conducting a research project is based on the research question and should be considered early in the process in order to avoid waste of resources and intellectual energy. This is sometimes difficult for a new investigator and they need guidance from their mentors.[4]

- Consider doing a pilot or proof of concept study to asses the feasibility;
- Consult a biostatistician early in the project in order to choose less costly design and common outcomes;
- Consider feasibility of enrolling the intended number of subjects from the population of your interest. Also, consider expanding your inclusion criteria and modifying exclusion criteria if it is difficult to enroll the intended number; and
- Consider cost of each element of the study design, research staff, and resources.

### Significance of making it interesting and relevant

An important question may not seem interesting the way it is presented. It is a challenge to present a research question clearly and engage the interest and attention of the reviewers. Research is too much work to not have a passion for what you are investigating. You will have more support for your study, and it will be easier to publish if the topic is novel and also interests your collaborators, colleagues, and the community at large. It is important to pursue a research question with a passion of getting the truth out of the matter.[5] This is how we all perceive research; commitment to a high-quality systematic and unbiased completion of an innovative project. If your question can explain a given problem while pointing toward a specific aspect which is missing then your project can get a great deal of support.

### Conducting literature review

The innovation of any research question is determined by a thorough literature search. Any replication of the study already existing in the literature is not worth repeating as it is. Depending upon the research question, sometimes the study can be replicated if your question approaches an existing problem in a refreshing way. This can be achieved by using a different populations, different techniques, new conceptual approaches, or linking two different studies in which outcomes did not solve the problem.[5] Once a preliminary question has been formulated, literature search should be done to find out what is known or unknown about the topic. The goal of the literature review is to determine what research has been conducted on the topic of interest? and how has it been conducted? and what are the gaps in the knowledge?. It is recommended to use PubMed, MedlinePlus, CINAHL, or Web of Science as the main search databases, but other databases can be used as well. PubMed clinical query is an easy and user-friendly database to search for evidence related to clinical practice. This also provides information to search MEDLINE by doing categorical searches, for example, therapeutic, diagnostic, etiological, and prognostic. The American College of Physicians (ACP) and clinical evidence from BMJ Publishing Group are excellent systems to find evidence on therapeutic questions. Other search engines such as OVID has a large selection of texts and journals which provides access to other databases such as Cochrane library in getting full text articles and systematic reviews. Gray *et al*. suggested *4 Ss* for literature review: *Systems*: use of comprehensive resources, *Synopses*: extracting high-quality studies and abstracts, Syntheses: systematic reviews, and *Studies*: original research studies.[6] In the hierarchy of evidence-based medicine, systematic reviews are considered the best method for evidence. Systematic reviews are rigorous methods of collecting and synthesizing the results of many high-quality studies. Conducting a thorough literature search also helps in finding information on the methodology, calculating the sample size, and also the type of analysis as we are looking to find a difference. This information is necessary to help structure a new study and to identify gaps in the knowledge base of the scientific community.

### Refining research question

A focused research question leads to a systematic planning of a research project. The difficulty in framing a research question is not due to the lack of ideas. The challenge is to transform a novel research question into a valid study design which is the next step in refining a research question.

## SUMMARY

Asking a well-formulated research question is a starting point in conducting a quality research project and in evidence-based clinical practice. The framework presented in this paper can be helpful for a clinician to formulate a question and search for an answer and for a researcher to develop a new research project. The classical approach is to identify a research question followed by a thorough literature search keeping in mind the PICO and FINER criteria. If it is a well-defined research question, it will lead to an appropriate study design and methodology. Discussing your research question with knowledgeable peers, department chair, mentor, and the biostatistician from the start will lead to the completion of a successful project. Other steps such as type and phase of the clinical trial, budget, informed consent, sites, resource constraints of both personnel and facilities, and timeline should also be considered while formulating a research question. We have introduced the concept of background and foreground questions and also the types of different questions that can arise (therapy, harm, diagnosis, and prognosis). We have described several strategies here while highlighting the major steps that will help investigators in framing a question with the goal of finding an answer based on evidence or initiation of a new research project. It is always good to focus on a single research question based on its relevance to patient’s health or one primary objective to drive the study design.[4] Once we have formulated our research question, we need to keep track of the progress toward finding an appropriate answer and then finally applying the results to a specific patient population. In short, a researchable question is what leads toward the facts rather than opinion[7] and is clearly linked to the overall research project goal.
